# Bioactivated Glucoraphanin Modulates Genes Involved in Necroptosis on Motor-Neuron-like Nsc-34: A Transcriptomic Study

**DOI:** 10.3390/antiox13091111

**Published:** 2024-09-14

**Authors:** Aurelio Minuti, Alessandra Trainito, Agnese Gugliandolo, Ivan Anchesi, Luigi Chiricosta, Renato Iori, Emanuela Mazzon, Marco Calabrò

**Affiliations:** 1 IRCCS Centro Neurolesi “Bonino-Pulejo”, Via Provinciale Palermo, Contrada Casazza, 98124 Messina, Italymarco.calabro@irccsme.it (M.C.); 2 Department of Food Quality and Nutrition, Research and Innovation Centre, Fondazione Edmund Mach (FEM), Via E. Mach 1, 38098 San Michele all’Adige, Italy; 3 Department of Medical, Oral and Biotechnological Sciences, University “G. D’Annunzio” Chieti-Pescara, 66100 Chieti, Italy

**Keywords:** glucosinolates, isothiocyanates, transcriptomic analysis, pathway analysis, oxidative stress, necroptosis

## Abstract

Research on bioactive compounds has grown recently due to their health benefits and limited adverse effects, particularly in reducing the risk of chronic diseases, including neurodegenerative conditions. According to these observations, this study investigates the activity of sulforaphane (RS-GRA) on an in vitro model of differentiated NSC-34 cells. We performed a transcriptomic analysis at various time points (24 h, 48 h, and 72 h) and RS-GRA concentrations (1 µM, 5 µM, and 10 µM) to identify molecular pathways influenced by this compound and the effects of dosage and prolonged exposure. We found 39 differentially expressed genes consistently up- or downregulated across all conditions. Notably, *Nfe2l2*, *Slc1a5*, *Slc7a11*, *Slc6a9*, *Slc6a5*, *Sod1*, and *Sod2* genes were consistently upregulated, while *Ripk1*, *Glul*, *Ripk3*, and *Mlkl* genes were downregulated. Pathway perturbation analysis showed that the overall dysregulation of these genes results in a significant increase in redox pathway activity (adjusted *p*-value 1.11 × 10^−3^) and a significant inhibition of the necroptosis pathway (adjusted *p*-value 4.64 × 10^−3^). These findings suggest RS-GRA’s potential as an adjuvant in neurodegenerative disease treatment, as both increased redox activity and necroptosis inhibition may be beneficial in this context. Furthermore, our data suggest two possible administration strategies, namely an acute approach with higher dosages and a chronic approach with lower dosages.

## 1. Introduction

In recent years, increased attention has been paid to bioactive compounds due to their distinct nutritional value, numerous health benefits, and relatively limited adverse effects [1]. Indeed, several epidemiological studies have highlighted the significant role of bioactive chemicals in reducing the risk of diseases, such as cancer, diabetes, stroke, heart disease, obesity, and so on [2,3].

The main mechanism underlying the beneficial properties of these natural molecules appears to be related to their antioxidant action [4,5]. Oxidative stress (OS) arises from an imbalance between the reactive species’ production and the organism’s antioxidant defenses [6]. OS has been the object of investigation in the scientific community, as ever-increasing data have demonstrated the pathological consequences of high OS status in humans [7,8,9]. Increased OS has been associated with a plethora of conditions, and in particular, is considered a major contributor to neurodegenerative diseases, as it can worsen neuronal damage and promote the progression of various conditions, such as Alzheimer’s disease (AD), amyotrophic lateral sclerosis (ALS), Parkinson’s disease (PD), and multiple sclerosis (MS) [5,10,11,12].

In this context, the properties of several natural compounds, including polyphenols, flavonoids, certain vitamins, and carotenoids, may have potential in improving brain function for their ability to neutralize free radicals, induce the secondary antioxidant response, reduce inflammation, and modulate the signaling pathways involved in neurodegeneration, which ultimately contribute to minimizing cognitive impairments and disease progression [12].

**Figure 1:**
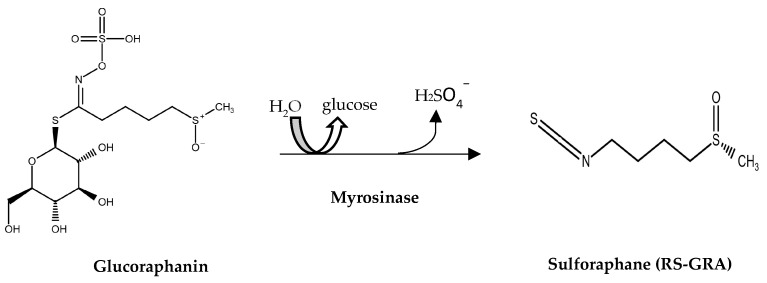
Hydrolysis reaction of GRA to RS-GRA exerted by Myr. The chemical structures of glucoraphanin and sulforaphane (RS-GRA) were obtained from the PubChem Compound Summary [14]. Details on the molecule’s properties can be found at https://pubchem.ncbi.nlm.nih.gov/compound/9548634 (accessed on 25 July 2024); Sulforaphane|C6H11NOS2|CID 5350—PubChem (nih.gov).

Vegetables, fruits, and whole grains are considered an important source of bioactive natural compounds. Of particular interest, diets abundant in cruciferous vegetables, such as cauliflower, broccoli, Brussels sprouts, and cabbage, have health-promoting effects due to their secondary metabolites, the glucosinolates (GSLs) [13]. GLSs are a class of naturally occurring organosulfur compounds; they are the precursors of isothiocyanates (ITCs) [13]. GLSs consist of a sulfur-bonded β-D-glucopyranose residue, a hydroxylamine sulfate ester, and a variable aglycon side chain that is derived from an α-amino acid (R-group). Depending on the specific amino acid from which the R-group originates, GLS can be classified into aliphatic (alanine, leucine, isoleucine, methionine, or valine), indole (tryptophan), and aromatic (phenylalanine, tyrosine) categories [13]. Natively, GLSs are biologically inactive compounds; they are activated through enzymatic hydrolysis, typically performed by a glycoprotein called myrosinase (Myr), also known as thioglucosidase glucohydrolase. The hydrolysis of GLSs results in a variety of biologically active compounds, including indoles, thiocyanates and isothiocyanates [13]. Sulforaphane (RS-GRA) (1-isothiocyanato-4-methylsulfinylbutane) is the hydrolyzed active compound of glucoraphanin (GRA) (Figure 1).

Several studies observed the antioxidant, anti-inflammatory, and antiapoptotic properties of this phytocompound and highlighted its potential neuroprotective action [15,16]. In detail, RS-GRA is known to be a powerful inducer of the nuclear factor erythroid 2-related factor 2-antioxidant response element (NRF2-ARE) pathway [17]. The activation of the NRF2-ARE pathway induces the transcription of many downstream products involved in protection against OS, including glutathione (GSH) peroxidase 1 (GPX1), NAD(P)H quinone dehydrogenase 1 (NQO1), heme oxygenase 1 (HMOX1), and the glutamate–cysteine ligase complex (composed of the GCLC and GCLM subunits), an important enzyme that controls GSH synthesis [18]. Furthermore, RS-GRA downregulates the expression of pro-inflammatory cytokines, such as interleukin-1β (IL-1β), interleukin-6 (IL-6), and tumor necrosis factor-α (TNF-α) by downregulating the nuclear factor-kappa-light-chain-enhancer of activated B cells (NF-κB), the main transcription factor involved in regulating cells’ responses to OS and inflammation [19]. Due to its properties, RS-GRA offers a promising approach for the prevention and treatment of several neurodegenerative diseases, such as AD, PD, and MS [10,11]. Indeed, in transgenic mouse models of AD, RS-GRA reduces the concentration of amyloid beta (Aβ) and phosphorylated tau (pTau) proteins, as well as their aggregation, thus ameliorating memory deficits [20].

Furthermore, using compounds with a low risk of adverse effects is particularly valuable in treating neurodegenerative diseases, which require chronic management [21]. The long-term use of medications often increases the risk of side effects and complicates treatment. However, employing such low-risk compounds as RS-GRA could help mitigate these challenges.

Given its potential, the use of RS-GRA might slow disease progression and ameliorate neurodegenerative-related symptoms, thus improving the quality of life for individuals affected by these debilitating conditions. Notably, literature data have shown RS-GRA neuroprotective properties and its importance in preventing neuronal cell death [22,23]. Whether cell death prevention is related to RS-GRA effects on OS or to is also related to RS-GRA regulation of different pathways is still under research.

In this context, continued research into the mechanisms of action and therapeutic potential of this natural compound may provide essential information to develop effective neuroprotective strategies. For these reasons, in this study, we investigated the biological processes triggered by this compound using next-generation sequencing (NGS) transcriptomic analysis. Specifically, we differentiated the spinal cord × neuroblastoma hybrid cell line (NSC-34) in the presence of all-trans retinoic acid (RA) [24]. We then examined the impact of RS-GRA on NSC-34 at various concentrations (1, 5, and 10 µM) and designed our experiments to also focus on prolonged effects (up to 72 h). This approach could help identify the core pathways involved in its long-term action and further characterize its mechanics.

## 2. Materials and Methods

### 2.1. Synthesis, Extraction, Purification, and Bioactivation of GRA

RS-GRA is unstable and poorly soluble in water, so rather than using the compound directly, we treated RA-differentiated NSC-34 cells with GRA together with homogeneous Myr. GRA isolation procedure was performed according to the protocol developed by CRA-CIN in Bologna, Italy [25]. Suba Seeds (Longiano, Italy) provided the Tuscan black kale seeds, which were initially ground into a fine powder and then defatted in hexane. The solvent was then removed, and the defatted solute was boiled with 70% ethanol (1:8 *w*/*v*) to prevent GL hydrolysis, as this procedure inactivates endogenous Myr enzyme. The solid residue was sorted out by centrifugation. The isolation of GRA was performed by one-step anion exchange chromatography, and then purified by gel filtration using an XK 26/100 column packed with Sephadex G10 chromatography media (GE Healthcare, Chicago, IL, USA), in an AKTA-FPLC system (GE Healthcare, Chicago, IL, USA).

Fractions were examined using HPLC to select the ones containing pure RS-GRA. HPLC analysis with 1H and 13C NMR spectroscopy was used to estimate the absolute purity of GRA, which according to the ISO 9167-1 method [26] was 99% (peak purity HPLC) and >95% weight basis (hydrated salt containing 1–2 equivalents of water). UV spectra and the molar extinction coefficient value of 6634 M^1^/cm^1^ at 225 nm were determined using a Varian Cary 300 Bio UV/vis spectrophotometer (Varian Medical Systems, Palo Alto, CA, USA). The selected fractions were then blended and lyophilized [27].

Myr enzyme was extracted from Sinapis alba L. seeds in accordance with Pessina et al. [28]. The enzymatic activity is 35 U/mL, and the enzyme was stored at 4 °C in a sterile saline solution at neutral pH until use. One Myr unit is defined as the amount of enzyme able to hydrolyze 1 μmol of sinigrin per minute at pH 6.5 and 37 °C [25]. A total of 1 mg of GRA powder was dissolved in phosphate-buffered saline (PBS) 1× (Sigma-Aldrich, Saint Louis, MO, USA). To allow the bioactivation of the phytochemical, we incubated GRA with Myr (0.64 U) for 1 h at 37 °C [29].

### 2.2. NSC-34 Cell Culture and Treatment

Neuroblastoma × spinal cord cells (NSC-34) were acquired by Cellutions Biosystems Inc., Cedarlane (Burlington, ON, Canada), and were maintained in DMEM high glucose, supplemented with 10% fetal bovine serum (FBS), 1% penicillin/streptomycin (P/S), and 1% L-Glutamine (L-Glu). All reagents were purchased from Sigma-Aldrich, Merck KGaA (Darmstadt, Germany). Cells were incubated at 37 °C in a moisturized atmosphere of 5% CO_2_.

### 2.3. Cell Differentiation and Treatment

NSC-34 cells were harvested onto 96-well plates (2500 cells/well), and 6-well plates (5 × 10^4^ cells/well) and were used for the MTT assay, transcriptomic analysis, and Western blot. One day after plating (D0), NSC-34 cell cultures were switched to DMEM/Ham’s F12 1:1, 1% FBS, 1% L-Glu, 0.5% P/S, and 1 µM/mL retinoic acid (RA) to induce differentiation. Subsequently, the cell population was allowed to differentiate for 5 days. Each type of experiment was performed on the same passage.

### 2.4. MTT Assay

To assess the possible effects of RS-GRA and Myr on the viability of NSC-34 cells, we performed the MTT assay, a colorimetric method for evaluating cell metabolic activity. NSC-34 cells were seeded in 96-well plates (2500 cells/well), and incubated at 37 °C for 24, 48, and 72 h with different GLs concentrations (from 0.5 μM to 10 μM of GRA). At the designed time points, the medium was replaced with a new one containing 0.5 mg/mL MTT (Sigma-Aldrich Merck KGaA (Darmstadt, Germany)) at 37 °C for 4 h. The obtained formazan crystals were dissolved in isopropanol (acidic) at 37 °C for 1 h. The optical density (OD) was evaluated by measuring absorbance at 570 nm through a spectrophotometer. Relative cytotoxicity was calculated as the percentage of viable cells compared to the control (CTRL) OD value of living cells (set as reference point: 100%).

### 2.5. RNA Extraction and cDNA Library Preparation

After treatment, the cells were harvested using a 0.25% trypsin–ethylenediaminetetraacetic acid (EDTA) solution (Sigma Aldrich, Saint Louis, MO, USA) and centrifuged (300× *g* for 5 min). Total RNA was extracted using the Maxwell^®^ RSC simplyRNA Cells Kit (Promega, Madison, WI, USA), according to manufacturer’s instructions. The library preparation was performed using the TruSeq RNA Exome Protocol (Illumina, San Diego, CA, USA) according to manufacturer’s instructions.

### 2.6. Transcriptomic Analysis

RNA-seq analysis for each concentration (1, 5, and 10 µM) and at different times (24 h, 48 h, and 72 h) of RS-GRA vs. CTRL was performed through next-generation sequencing (NGS) technology. The Illumina NextSeq 550 Series was used for the sequencing step. The quality of the raw data was confirmed by the fastQC tool, and then Trimmomatic version 0.40 (Usadel Lab, Aachen, Germany) was used to remove the adapters and prune the bases of low quality [30]. The reads were aligned to the reference genome of the mouse organism GRCm39 and arranged using Spliced Transcripts Alignment to a Reference (STAR) RNA-seq aligner 2.7.10a (New York, NY, USA) [31]. For each genomic area, the reads found were counted using the htseq-count program (European Molecular Biology Laboratory (EMBL), Heidelberg, Germany) [32]. The R programming language and the Bioconductor tool DESeq2 were used to identify the genes that varied in a statistically significant way between the CTRL and treated NSC-34 [33]. Genes were considered differentially expressed (DEGs) between the two groups if they had an adjusted *p*-value < 0.05.

Statistical correction was performed with the Benjamini–Hochberg method. A total of 9 lists of DEGs were obtained from the comparisons (1 µM RS-GRA 24 h vs. CTRL 24 h, 5 µM RS-GRA 24 h vs. CTRL 24 h, 10 µM RS-GRA 24 h vs. CTRL 24 h, 1 µM RS-GRA 48 h vs. CTRL 48 h, 5 µM RS-GRA 48 h vs. CTRL 48 h, 10 µM RS-GRA 48 h vs. CTRL 48 h, 1 µM RS-GRA 72 h vs. CTRL 72 h, 5 µM RS-GRA 72 h vs. CTRL 72 h, and 10 µM RS-GRA 72 h vs. CTRL 72 h).

### 2.7. Statistical Analysis

Statistics for survival analyses were performed with GraphPad Prism version 10.1 software (GraphPad software, La Jolla, CA, USA). To ensure data consistency, 5 biological replicates were used; the highest and lowest values were excluded, and the mean was calculated from the remaining 3 replicates for each condition. Results are expressed by the mean ± SD. Multiple comparisons were tested with one-way ANOVA test and corrected by Bonferroni post hoc test. An adjusted *p*-value ≤ 0.05 was considered statistically significant.

### 2.8. DEGs Filtering Process

After obtaining the lists of significant DEGs for each of the 9 tested comparisons (please refer to Section 2.6), we applied a rigorous filtering process based on their behavior across different conditions to ensure the reliability and consistency of the selected DEGs. Specifically, we selected DEGs that showed consistent expression patterns across multiple concentrations and time points tested. The rationale for the concentration-based filtering was that if a cell is exposed to a specific compound, its molecular machinery is expected to respond consistently, albeit with potential variations in the level of dysregulation. The consistency across concentrations may be helpful to select the core genetic elements linked to the cellular response to the compound. According to this, we filtered out DEGs that showed different expression behavior at the tested concentrations (i.e., DEGs upregulated at 5 µM but downregulated at 10 µM were excluded).

Regarding the time-based filtering, we focused on selecting genes that remained dysregulated across various time points. This approach was based on the fact that we are mainly interested in identifying the stable effects of the compound. Therefore, we wanted to highlight genes exhibiting sustained dysregulation over time. According to this, we filtered out DEGs that showed inconsistent expression behavior at different times (i.e., DEGs upregulated at 48 h but downregulated at 72 h were excluded).

By implementing these criteria, we aimed to ensure that the selected DEGs have a higher probability of being core elements linked to the compound action. As we focused on consistency at different times and concentrations, we did not put any constraints on fold-change.

### 2.9. Bioinformatic Analyses

The DEGs selection (after the filtering step) was further investigated to identify biological processes significantly enriched in DEGs, and to evaluate if said processes were inhibited or activated. To perform this, we applied a signaling pathway impact analysis (SPIA), an approach combining classical enrichment analysis with another algorithm that evaluates the perturbation on a given pathway under a given condition. In detail, the perturbation score is evaluated through a bootstrap procedure, which ultimately assesses the significance of the observed score for each pathway [34]. To select strongly perturbed pathways, we filtered out only the results with a perturbation score (tA) < $\left|2\right|$.

The graphite package v.1.50.0 [35,36] and the SPIA package v.2.56.0 [34] in R v.4.4.1 environment were used to perform SPIA analysis. Pathways were retrieved from KEGG (https://www.kegg.jp/kegg/, accessed on 25 June 2024), Pathbank (https://pathbank.org/, accessed on 25 June 2024), and Wikipathways (https://www.wikipathways.org/, accessed on 25 June 2024) public databases. Pathways were considered significantly perturbed (either inhibited or activated) if the related adjusted *p*-value was <0.05. To decrease the risk of false positives, the *p*-value was corrected through the family-wise error rate (Bonferroni) correction.

Plots were produced in the Python v.3.12.3 environment, using the matplotlib v.3.8.4 [37] and seaborn v.0.13.2 [38] packages.

### 2.10. Western Blot Analyses

NSC-34 cells were harvested at the end of 24, 48, and 72 h treatments, and proteins were extracted using the NE-PER™ Nuclear and Cytoplasmic Extraction Reagents (Thermo Scientific™, Waltham, MA, USA) according to the instructions. Proteins’ concentration was evaluated with a Bradford assay (Bio-Rad, Hercules, CA, USA). A total of 25 µg of proteins were heated at 95 °C for 5 min. Then, proteins were loaded, resolved by sodium dodecyl sulfate–polyacrylamide gel electrophoresis (SDS-PAGE), and transferred onto a polyvinylidene difluoride (PVDF) membrane (Immobilon–P, Millipore, Burlington, MA, USA). Next, 5% skimmed milk dissolved in Tris-buffered saline (TBS) was used to block membranes at room temperature for 1 h, followed by the overnight incubation at 4 °C. The antibodies used were anti-Ripk3 (dilution 1:2000), anti-Mlkl (dilution 1:1500), anti-Ripk1 (dilution 1:1500), and anti-Nrf2 (dilution 1:500). Additionally, we used anti-Sod1 (dilution 1:1250) to further evaluate the effect of RS-GRA on OS. After washing with TBS 1X, membranes were incubated with HRP-conjugated anti-rabbit antibody (1:2000; Santa Cruz Biotechnology Inc., Dallas, TX, USA) for 1 h at room temperature. After acquisition, membranes were stripped with Restore Western blot buffer (Thermo Scientific, Meridian, Rockford, IL, USA) and then incubated with glyceraldehyde-3-phosphate dehydrogenase (GAPDH) HRP-conjugated antibody (1:1000; Cell Signaling Technology, Danvers, MA, USA) for the cytoplasmatic compartment and Lamin B1 (1:500; Cell Signaling Technology, Danvers, MA, USA) for nuclear extracts, used as a loading control.

Protein bands were visualized using the Immobilion Forte Western HRP Substrate (Millipore Corporation, Burlington, MA, USA). Protein band acquisition was performed on the ChemiDoc™ XRS + System (Bio-Rad, Hercules, CA, USA). Protein bands were quantified through ImageJ 1.54 software. The original uncropped membranes are available in the Supplementary Materials.

## 3. Results

### 3.1. MTT Assay

**Figure 2:**
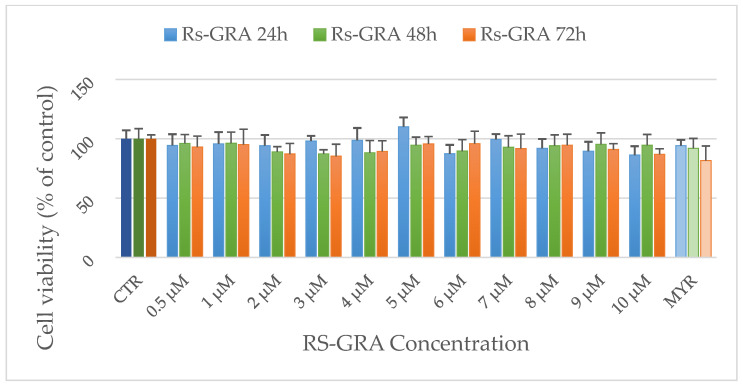
Cell viability tested with MTT assay in differentiated NSC-34 cells treated with RS-GRA at different concentrations (0.5–10 µM) after 24 h, 48 h, and 72 h. Results are normalized against CTRL and expressed as the mean ± SD. There were five biological replicates per condition. One-way ANOVA and a Bonferroni post hoc test showed no significant differences (*p*-value < 0.05) between treated cells and CTRL. Darker and lighter hues represent Controls and Myrosinase-only wells, respectively.

The spectrophotometric absorbance resulted from the MTT assay did not highlight any significant change after RS-GRA treatment at any tested concentration. Furthermore, exposure to Myr alone did not affect cell viability (Figure 2). According to these results, we concluded that the compound is not toxic at the tested dosages. Therefore, we performed transcriptomic analysis using RS-GRA at 1 µM, 5 µM, and 10 µM to encompass the range of tested concentrations.

### 3.2. Differential Expression Analysis (DEA)

**Figure 3:**
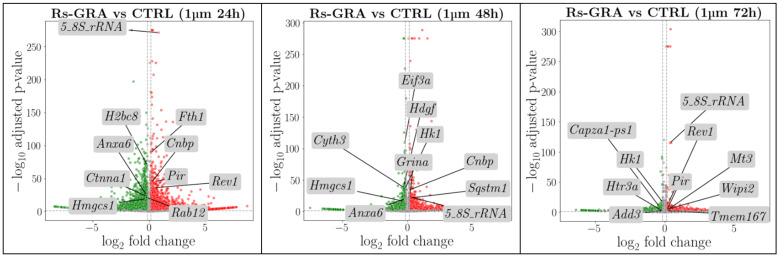

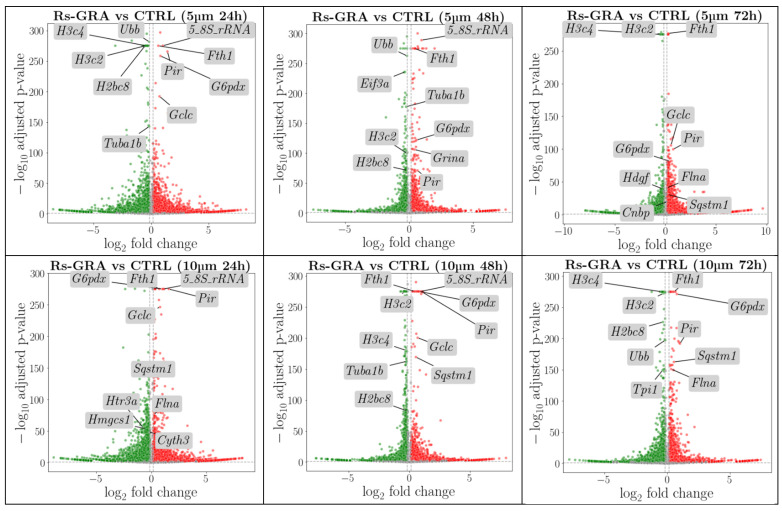
In the volcano plot, we report the log_2_ fold-changes and *p*-values of all the genes explored in the DEA for each comparison. The line that intercepts the y axis is related to our threshold of significance of 0.05; all the genes above this line are considered to be differentially expressed. The x axis reports the log_2_ fold-change that discriminates up- and downregulated DEGs defined by a red or green color, respectively. In the figures, we also report the top 10 genes that survived DEG selection.

DEA performed between treated and not treated cells at each concentration and time resulted in nine sets of genes whose expression was compared in the two conditions (RS-GRA treated vs. CTRL) investigated. We considered as upregulated all the DEGs that assumed a statistically significant expression increase in the treated group compared to CTRL, while the downregulated DEGs were the ones with a decreased expression in treated cells compared to CTRL. The statistical significance was adjusted according to the Benjamini–Hochberg method. Volcano plots summarizing the results of the DEA analyses are reported in Figure 3.

### 3.3. DEGs Filtering and Selection

**Figure 4:**
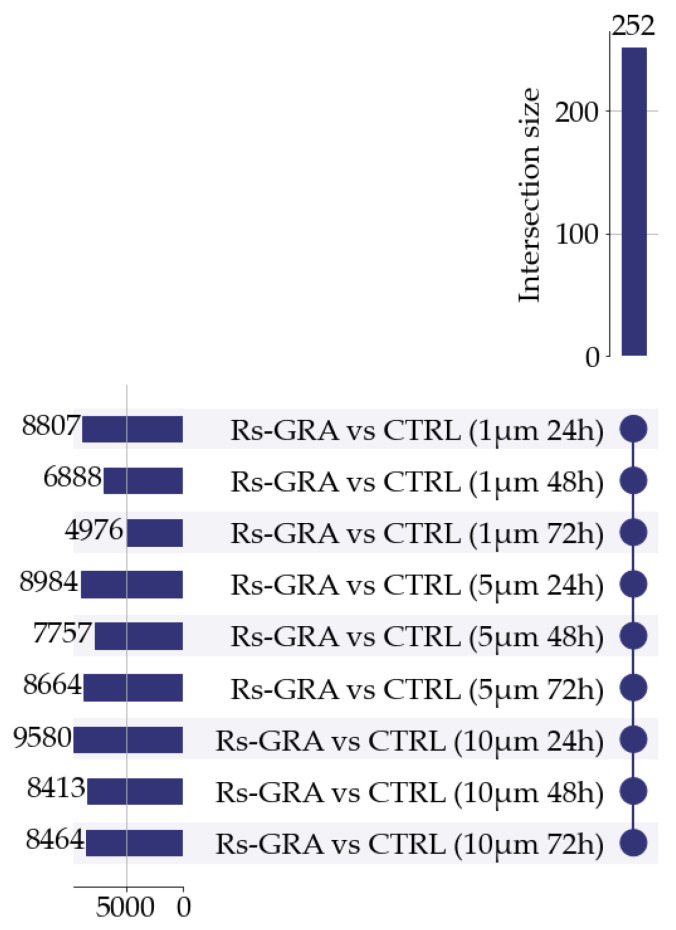
In the upset plot, we summarize all the DEGs that are consistently dysregulated in all investigated conditions. On the upper bar plot, we report the number of DEGs shared for each intersection considered (as indicated in the lower half of the plot). The bar plot on the left reports the DEGs resulting from each comparison. Only intersections with a size ≥ 9 are shown.

To identify DEGs consistently dysregulated by treatment at all concentrations and times, we applied a filtering process based on the behavior emerging from the nine DEA analyses. From the starting sets of DEGs ranging from 4976 to 9580 genes, we retained 252 DEGs that resulted dysregulated in all comparisons. Figure 4 summarizes the details.

Furthermore, from the list of 252 elements, we eliminated all DEGs that varied their expression behavior across the tested conditions (e.g., genes that were upregulated in one comparison and downregulated in another). This final selection resulted in 39 DEGs consistently upregulated or downregulated in all the comparisons (*5_8S_rRNA*, *Add3*, *Anxa5*, *Anxa6*, *Astn1*, *Capza1-ps1*, *Chpf2*, *Cnbp*, *Ctnna1*, *Cyth3*, *Eif3a*, *Flna*, *Fth1*, *G6pdx*, *Gclc*, *Glul*, *Gramd1a*, *Grina*, *H2bc8*, *H3c2*, *H3c4*, *Hdgf*, *Hk1*, *Hmgcs1*, *Htr3a*, *Mcm7*, *Mt3*, *Pir*, *Psat1*, *Rab12*, *Rev1*, *Rom1*, *Sqstm1*, *Tmem167*, *Tmem33*, *Tpi1*, *Tuba1b*, *Ubb*, and *Wipi2*). Notably, several of these DEGs play critical roles in managing OS and in cell death pathways. Genes, like Fth1 (Ferritin Heavy Chain 1) and G6pdx (Glucose-6-Phosphate Dehydrogenase X-Linked), play key roles in OS management. Fth1 helps control oxidative damage by sequestering iron, thus preventing the formation of harmful reactive oxygen species (ROS), while G6pdx produces NADPH, a critical cofactor for antioxidant defense systems. Similarly, Gclc (Glutamate–Cysteine Ligase Catalytic Subunit) is essential for synthesizing GSH, a major cellular antioxidant that neutralizes ROS and protects cells from oxidative damage. Mt3 (Metallothionein 3) also contributes to OS defense by binding metal ions and reducing metal-induced oxidative damage. On the other hand, some genes, such as Sqstm1 (Sequestosome 1) are involved in regulating cell death pathways. Sqstm1 plays a multifunctional role in autophagy and protein degradation, processes that are vital for responding to cellular stress and regulate programmed cell death, highlighting their significance in maintaining cellular homeostasis and preventing disease.

### 3.4. Signaling Pathway Impact Analysis (SPIA)

**Figure 5:**
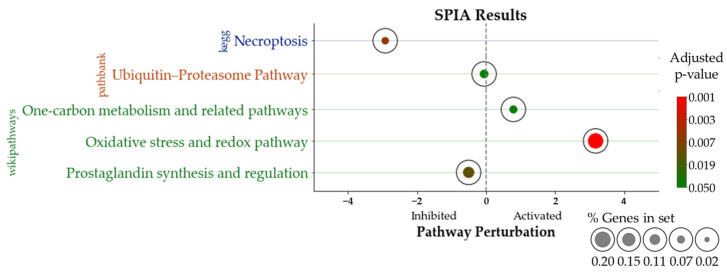
In the plot, we report the pathways significantly enriched in DEGs that also showed a significant perturbation according to SPIA results. On the lefthand side, the pathways inhibited by RS-GRA treatment are shown (necroptosis, ubiquitin–proteasome pathway, and prostaglandin synthesis and regulation); of these, only necroptosis showed a perturbation score(tA) higher than |2| (necroptosis tA: −2.95). On the righthand side, the pathways activated by RS-GRA treatment are reported (one-carbon metabolism and related pathways, oxidative stress and redox pathway); of these, only the oxidative stress and redox pathway showed a tA higher than |2| (OS and redox pathway tA: 3.17). Bubbles hue indicates the adjusted *p*-values, while size is related to the ratio of DEGs/total genes within the pathway under investigation.

The 39 DEGs resulting from the filtering process were used as the input for the SPIA. The aim of this analysis was to identify potential pathways related to the DEGs under investigation and to evaluate the level of perturbation induced by these dysregulated elements in such pathways. This analysis was based on multiple public datasets collecting pathway data for mice, namely KEGG, Pathbank and Wikipathways. The SPIA resulted in the identification of five pathways correlated with the dysregulated genes. Of these, the KEGG pathway of necroptosis was particularly inhibited, with a tA of −2.95, and the Wikipathways of “Oxidative stress and Redox pathway” were highly activated, with a tA of 3.17. Other pathways (ubiquitine–proteasome pathway, one-carbon metabolism and related pathway, and prostaglandin synthesis and regulation) showed significant associations with the DEGs under analysis; however, their perturbation score did not reach the threshold (please refer to Section 2.9). Details of the results obtained from SPIA are reported in Table 1, in Figure 5, and in Supplementary Figure S1.

### 3.5. Western Blot Analysis

**Figure 6:**
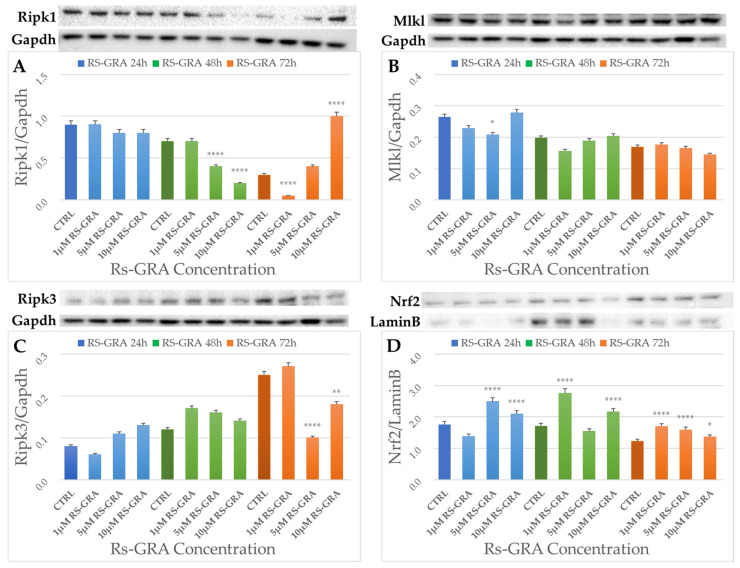
Here, we report proteins levels at all concentrations and all time-steps (bar plots) and the bands from WB membranes. (**A**) Ripk1 concentration at the cytosolic level compared to the housekeeping protein Gapdh. A significant decrease in protein expression compared to controls was highlighted at the later time-steps (48 h at 5 µM and 10 µM dosages and 72 h at 1 µM). Interestingly, we detected a drastic increase in this protein expression at 72 h at the 10 µM dosage. (**B**) Mlkl concentration at the cytosolic level compared to the housekeeping protein Gapdh. A significant decrease in protein expression compared to controls was highlighted only at the 5 µM at 24 h. (**C**): Ripk3 concentration at the cytosolic level compared to the housekeeping protein Gapdh. For Ripk3, a significant decrease in protein expression compared to controls was highlighted at the last time-step (72 h) at 5 µM and 10 µM dosages. (**D**) Nrf2 concentration at the nuclear level compared to the housekeeping protein Lamin B. A significant increase in protein expression compared to controls was observable at multiple time-steps and multiple dosages. The original membranes are included as Supplementary Materials (Supplementary Figures S2–S5). Asterisks (*) indicate *p*-value: * *p* < 0.05; ** *p* < 0.01; **** *p*< 0.0001, respectively.

To assess the results obtained from transcriptomic analyses on the potential cytoprotective effects of RS-GRA on NSC-34 cells, we evaluated protein levels of Ripk1, Ripk3, and Mlkl (necroptosis) and Nrf2 (oxidative stress and redox pathway), as reported in Figure 6. OS status was also confirmed by measuring Sod1 protein concentration. Details of WB results are available in the Supplementary Materials. The choice of these proteins was based on the rationale that these elements are the main representatives of the enriched pathways.

**Figure 7:**
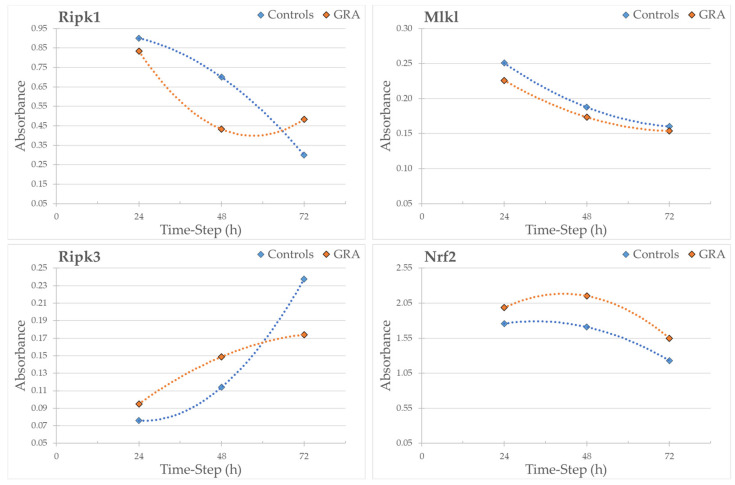
Here, we report the overall trend of proteins’ levels at the three time-steps (24 h, 48 h, and 72 h). Each point corresponds to the mean protein expression (normalized by the housekeeping protein expression) of the three dosages for each time-step. In orange, the protein expression levels from treated cells are reported. In blue, the non-treated controls are shown.

**Figure 8:**
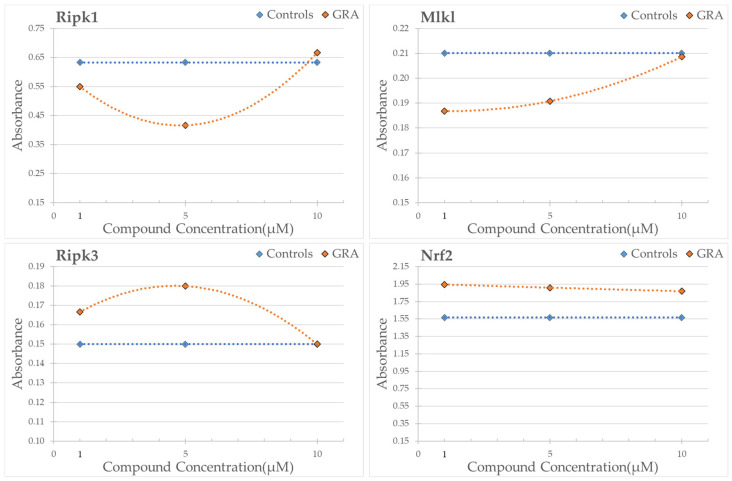
Here, we report the overall trend of proteins’ levels at the three tested concentrations (1 µM, 5 µM, and 10 µM) irrespective of the time-steps. Each point corresponds to the mean protein expression (normalized by the housekeeping protein expression) of the three time-steps for each dosage. In orange, the protein expression levels from treated cells are reported. In blue, the non-treated controls are shown.

Ripk3 levels decreased in a statistically significant way after 72 h of treatment at concentrations of 5 and 10 µM RS-GRA compared to the control. Mlkl was statistically downregulated at 5 µM concentration after 24 h RS-GRA treatment. Ripk1 decreased after 48 h of RS-GRA treatment at 5 and 10 µM, and it was downregulated at the 1 µM concentration. On the contrary, Ripk1 was upregulated at a concentration of 10 µM at 72 h. Nrf2 increased after 24 h at concentrations of 5 and 10 µM, after 48 h at 1 and 10 µM, and after 72 h at all concentrations. Data on Sod1 expression indicated an early increase in this protein (24 h and 48 h) followed by a sharp decrease (72 h). Details are reported in (Supplementary Figures S6–S8). Finally, the overall trend of proteins levels is reported in Figure 7 and Figure 8.

## 4. Discussion

In recent years, RS-GRA has been the object of active research for its protective potential, as shown in several in vitro models [39,40]. As RS-GRA is able to cross the blood–brain barrier, this compound has a recognized potential to be directly used to protect against brain damage [11,40,41]. Due to these properties, we performed a transcriptional analysis on differentiated NSC-34 cells exposed to the compound (different concentrations and time-steps) and compared it to untreated differentiated NSC-34 cells to highlight genes whose expression is influenced by RS-GRA.

NSC-34 cells are commercially available, and they are obtained by the hybridization of embryonic spinal cord cells and neuroblastoma cells from mice. NSC-34 cells are usually used to study motoneuron-like behavior [42]. RA in combination with serum deprivation induces the differentiation of NSC-34 cells. This induces the expression of several neuron-like properties, so they are used as an appropriate in vitro model to study the impact of neurodegenerative diseases, and in particular in the pathophysiology of motor neurons [42,43]. Differentiation of neuronal cells is useful to investigate biochemical and morphological characteristics as well as the injury susceptibility of neurons.

The first step of our study was to assess the effects of RS-GRA on cell viability and metabolic activity using the MTT assay. The results indicated that this compound does not negatively affect cell viability; therefore, we concluded that RS-GRA does not have cytotoxic effects at the tested dosages (please refer to Figure 2). After ensuring the safety of RS-GRA, we performed an NGS analysis on the transcriptome to evaluate the genes’ expression behavior in the two conditions (treated vs. not treated) and pinpoint molecular pathways directly influenced by this compound (SPIA analysis). To heighten the stringency of our findings and to reduce the risk of false positives in the SPIA analysis, we took into consideration only the DEGs obtained from the nine comparisons that showed consistent dysregulation across the different conditions (different concentrations and times). This step was pivotal to increase the likelihood of pinpointing core gene clusters influenced by the compound. The set of 39 DEGs retained was, thus, used in the analysis to evaluate pathways associated with the compound and their overall perturbation.

The results we obtained highlighted the enrichment and significant perturbation of the oxidative stress and redox pathway (Wikipathways) and the necroptosis pathway (KEGG), as described in the Results section (Figure 5). This implies that these two processes likely play a key role in the compound’s functions.

We then investigated the behavior of all the elements within these pathways according to our transcriptomic data.

**Figure 9:**
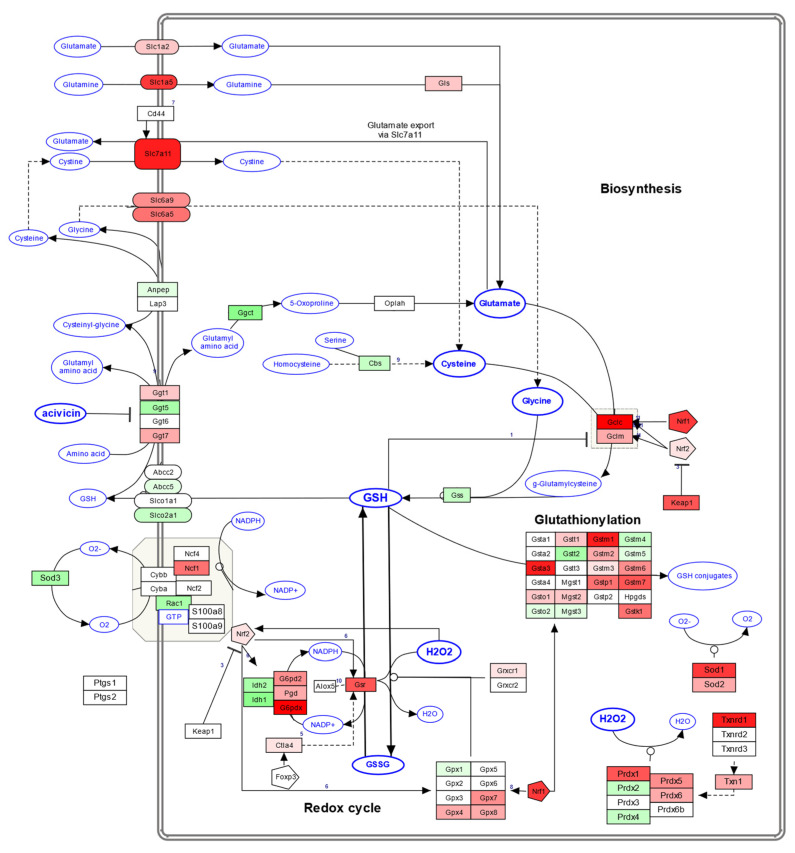
Here we report the oxidative stress and redox pathway from Wikipathways. DEGs from our data are reported in red (upregulated) or green (downregulated) according to their expression behavior. The figure was obtained from Wikipathways website and colored based on our data. The color intensity is based on a +9 to −9 scale that summarizes in how many conditions each transcript was significantly dysregulated (from upregulated at all time-steps and dosages to downregulated at all time-steps and dosages).

Regarding the antioxidant stress response, it is not surprising that we found the pathway activated, as the rich literature data confirm the antioxidant properties of RS-GRA [22]. In detail, we found several DEGs associated with the antioxidant response pathway, including *G6pd* and *Gclc*, which were among the 39 DEGs selected, and *Gpx4*, *Nfe2l2*, *Oplah*, *Prdx1*, *Sod1*, *Sod2*, *Slc7a11*, and *Txn1*, which were also partially dysregulated according to our transcriptomic analysis. In Figure 9 we report the oxidative stress and redox pathway from Wikipathways, highlighting the DEGs obtained.

Of these elements, the most important in the context of OS response is likely Nrf2, encoded by the *Nfe2l2* gene. This protein is usually found in the inactive state bound to the repressor protein Kelch-like ECH-associated protein 1 (Keap1) [44,45]. When activated, Nrf2 dissociates from its repressor, translocases into the nucleus, and modulates gene expression by reacting with the promoter region of antioxidant-responsive elements (AREs) [45].

Other DEGs likely implicated in the redox function included *Txn1*, which is involved in the reversible S-nitrosylation of cysteine residues and thereby inhibits caspase 3 [46]; *Gclc* is the first rate-limiting enzyme of GSH synthesis. It participates in the first and rate-limiting step in GSH biosynthesis, and *Gclc* dysregulation was found in neurological disorders [47]. *Prdx1* encodes a member of the peroxiredoxin family of antioxidant enzymes, reducing hydrogen peroxide and alkyl hydroperoxide. It plays an antioxidant protective role in cells by detoxifying peroxides [48]. *Slc7a11* mediates the exchange of extracellular anionic L-cysteine and intracellular L-glutamate across the cellular plasma membrane. This provides L-cystine for the maintenance of the redox balance between extracellular L-cystine and L-cysteine and for the preservation of the intracellular levels of GSH that is essential for cells’ protection from OS. In addition, it mediates the import of L-kynurenine, leading to the anti-ferroptosis signaling propagation required to maintain L-cystine and GSH homeostasis [49]. We also found upregulation of the *Oplah* gene, which catalyzes the conversion of 5-oxo-L-proline to L-glutamate [50]. 5-Oxoproline has been shown to induce OS in rat brain tissue, human embryonic derived cardiomyocytes, and rat cardiomyocytes [51,52,53]. RS-GRA also upregulated *Sod1* and *Sod2* gene expression. They encode for two isozymes of the superoxide dismutase (SOD), respectively, which belong to a class of oxidoreductase and are responsible for eliminating free superoxide radicals, converting them into molecular oxygen and hydrogen peroxide [54]. Brain SODs are reported to play a pivotal role in regulating the redox balance and protecting the brain from oxidative damage [54]. Neurodegenerative diseases, such as ALS, AD and PD, are characterized by increased OS and reduced levels of antioxidant enzymes, including SOD [54].

It is important to note that we also observed the upregulation of the *Gpx4* gene at all concentrations. This gene catalyzes the reduction of hydrogen peroxide, lipid hydroperoxides, and organic hydroperoxides, actively protecting cells against oxidative damage [55].

**Figure 10:**
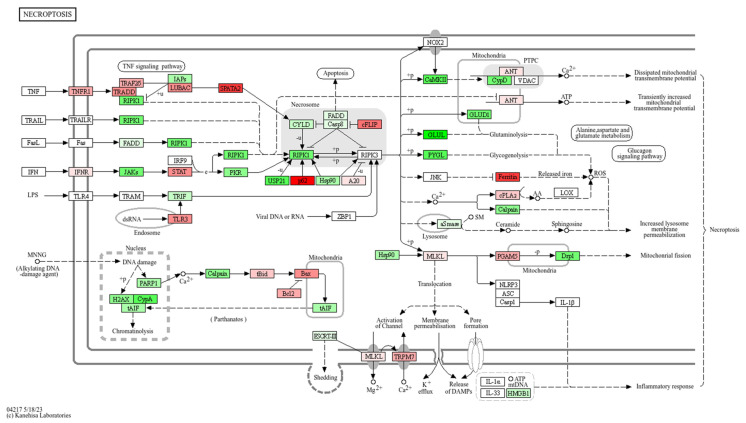
Here we report the necroptosis pathway from KEGG. DEGs from our data are reported in red (upregulated) or green (downregulated) according to their expression behavior. The figure was obtained from KEGG website and colored based on our data. The color intensity is based on a +9 to −9 scale that summarizes in how many conditions each transcript was significantly dysregulated (from upregulated at all time-steps and dosages to downregulated at all time-steps and dosages).

While the antioxidant properties of RS-GRA are well documented in the literature, fewer data are available regarding its capacity of inhibiting necroptosis. Our SPIA analysis highlighted this pathway as being significantly perturbed (inhibited). According to our results, the treatment with RS-GRA downregulated the expression of several necroptosis-associated genes, including *Fth1*, *Glul*, and *Sqstm1*, which were among the 39 DEGs selected, while *Cyld*, *CypD*, *Ifnr*, *Nlrp3*, *Mlkl*, *Pygl*, *Ripk1*, *Slc25a4s*, and *Vdac* were also partially dysregulated in our transcriptomic analysis (Figure 10).

Necroptosis is an alternative form of programmed cell death (PCD) that leads to cell membrane rupture and inflammation [56,57]. It is induced by the stimulation of tumor necrosis receptor (TNFR1), Fas cell surface death receptor (Fas), and/or by the activation of Toll-like receptors (TLRs). These receptors stimulate the receptor-interacting serine/threonine protein kinase 1 (RIPK1) [58]. This allows RIPK1 to activate the kinase RIPK3 within a cytoplasmic high-molecular-weight complex named the necrosome. Then, RIPK3 activates the pseudo-kinase MLKL, which causes the lysis of the plasma membrane [59,60]. Of particular interest, necroptosis is strongly linked with the response to OS, as it has been observed that excessive presence of ROS may trigger this type of PCD. Indeed, ROS have long been considered a driving force for RIPK1-dependent necroptosis [61].

Necroptosis has been linked to several neurodegenerative disorders. Furthermore, recent literature data have demonstrated neuroprotective effects associated with the inhibition of this pathway, which could be targeted for the treatment of neurodegenerative diseases [62,63]. In ALS, necroptosis is strictly involved in motor neuron degeneration [64]. In the post-mortem examination of brains from human AD patients, an abundant expression of MLKL compared to brains of healthy controls was found. Moreover, necroptosis is able to exacerbate cognitive deficits in the APP/PS1 mouse model of AD. The inhibition of RIPK1 through necrostatin-1, has been proven to reduce neuronal death, attenuating the formation of insoluble Aβ plaques and hyperphosphorylated tau, ultimately ameliorating cognitive dysfunction [65,66]. Finally, in preclinical models of PD, downregulation of MLKL, RIPK1, and RIPK3 exerted neuroprotective effects, with improved motor performance and decreased dopaminergic neuron degeneration [67].

The *Sqstm1* gene encodes for a multifunctional protein (p62) that binds ubiquitin, regulates the activation of the nuclear factor kappa-B (NF-kB) signaling pathway, and is induced by Nrf2 [68]. This element is also involved in the regulation of Ripk1. Interestingly, several neurodegenerative diseases are characterized by low levels of p62 [69], and our results highlighted an upregulation of this element induced by RS-GRA.

Of the other DEGs related to necroptosis, the *Cyld* gene encodes a cytoplasmic protein that is involved in NF-κB activation, contributing to the inflammatory response and cell survival. Interestingly, it has been shown that downregulation of *CYLD* could be neuroprotective [70,71]. Nlrp3 is an upstream activator of NF-κB signaling, playing a pivotal role in the regulation of inflammation, apoptosis, and the immune response. NLRP3 activation mediates inflammasome activation in response to defects in membrane integrity [72]. The *Glul* gene belongs to the glutamine synthetase family and catalyzes the synthesis of glutamine from glutamate and ammonia, regulating the levels of toxic ammonia, and converts neurotoxic glutamate to glutamine. Upregulation of *Glul* is linked to neurotoxicity and neurodegeneration [73].

To further confirm the involvement of the two pathways highlighted by our transcriptomic data, we proceeded to test protein expression through WB analysis. As we tested all the nine conditions (concentrations and times), we also obtained information on the expression trend of the selected elements.

As a known biomarker of redox activity, we chose to evaluate Nrf2 to confirm the oxidative stress and redox pathway activation, although in our transcriptomic data it was dysregulated in only seven of the nine conditions. According to WB data on the nuclear fraction of proteins, Nrf2 is upregulated in cells treated with RS-GRA compared to untreated cells (please refer to Figure 6). It should be noted that the increase in this protein in the nuclear compartment is a sign of increased activity, as Nrf2 is a transcription factor.

Interestingly, we evidenced a decreasing, albeit slight, trend in Nrf2 protein concentration at RS-GRA higher dosages after prolonged exposure. While overall this protein concentration resulted significantly increased compared to untreated cells, this decrease was a phenomenon we also detected in the other tested proteins, including Sod1, whose decrease in concentration at 72 h was more evident (Supplementary Figure S6).

To confirm the inhibitory effect of RS-GRA on necroptosis, we assessed the levels of Ripk1, Ripk3, and Mlkl proteins. Similarly to Nrf2, the mRNA of these protein did not become dysregulated at all times and conditions according to the transcriptomic data. Nevertheless, we chose these protein as they are key elements of the necroptosis pathway.

Although less evident than the effect on Nrf2, we confirmed a reduced expression of such proteins in treated cells. Interestingly, this effect appears more evident at the later time-steps, as summarized in Figure 7, suggesting a long-term/delayed action of this compound. It should be noted that Ripk1 after 72 h at 10 µM showed the opposite effect of what we expected: we observed a moderate to high increase in this protein in the treated cells. This result may be correlated with 72 h exposure and a high dosage. Indeed, prolonged exposure to higher dosages seem to invert the trend of RS-GRA for Mlkl and Ripk1, suggesting an opposite effect on necroptosis (please refer to Figure 8), which stresses the delicate equilibrium of this pathway control.

**Figure 11:**
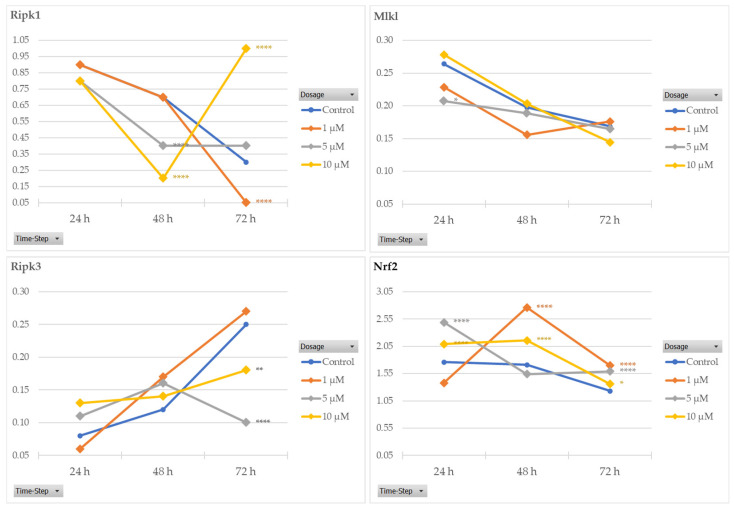
Here, we report the overall trend of proteins’ concentrations at the three tested concentrations (1 µM, 5 µM, and 10 µM) at the different time-steps (24 h, 48 h, and 72 h). Each point corresponds to the protein expression normalized by the housekeeping protein expression (GAPDH of necroptosis genes and Lamin B for Nrf2) of the three time-steps for each dosage. Protein expressions in untreated cells are reported in blue, while the different dosages (1 µM, 5 µM, and 10 µM) are reported in orange, gray, and yellow, respectively. Asterisks (*) indicate *p*-values: * *p* < 0.05; ** *p* < 0.01; **** *p* < 0.0001, respectively.

A deeper visualization of protein expression trends (Figure 11) shows how Ripk1 concentration is decreased compared to control at 48 h after exposure to 5 µM and 10 µM. Prolonging the exposure to 72 h trigger the opposite effect, normalizing protein expression at 5 µM and drastically increasing it at 10 µM compared to untreated cells. The 1 µM dosage instead appears to not be enough to trigger a Ripk1 decrease, which is reached after prolonged exposure (72 h). These data suggest two potential modalities of administration, one acute at higher dosages, and one chronic at lower dosages. Nevertheless, it should be stressed that the role of Ripk1 is complex, as pro-survival properties have also been attributed to this protein [61]. Its pro-survival role is achieved via its ability to induce NF-κB-dependent expression of anti-apoptotic genes [61]. As such, we cannot exclude (1) the presence of more complex mechanisms through which the cell increases Ripk1 production and/or decreases its degradation in an attempt to restore its functions; (2) the presence of potential protective effects associated to its increase. Further studies specifically aiming to evaluate the activation/inhibition of necroptosis would be needed to better evaluate the specific role of Ripk1 in this context.

Regarding Ripk3, this protein also appears to have a trend similar to Ripk1, although less evident, for the 10 µM dosage at 72 h exposure. The max difference in the Ripk3 protein concentration compared to untreated cells is achieved after 72 h using 5 µM RS-GRA.

As said before, no significant changes can be appreciated for Mlkl, while Nrf2 expression overall is increased in treated cells, albeit with a slight decrease in protein expression after prolonged exposure (72 h) to higher dosages.

Overall, due to the potential inhibitory properties of RS-GRA on necroptosis, and the existing link between necroptosis and such neurodegenerative conditions, the compound may provide beneficial effects for symptom management. Also, while some associations of RS-GRA with the general apoptotic mechanism are present in the literature, to the best of our knowledge, this is the first time that an anti-necroptotic action has been described for RS-GRA.

Interestingly, necroptosis and OS are linked and influence each other; the action of RS-GRA on both processes should have a positive effect on cell survival, as it should confer protection against ROS species, molecules commonly found elevated in many pathological conditions, in particular as a consequence of inflammatory states [74]. These molecules are also known to have high damage potential for cell organelles and macromolecules, including DNA [74,75]. Also, the inhibition of necroptosis would likely reduce cognitive symptoms related to neurodegenerative process, as described in the literature [76,77].

Based on the diverse effects observed at different dosages and time points, we can infer that the action of RS-GRA is influenced by both dosage and exposure duration, especially affecting the necroptosis pathway. In particular, high dosages and excessive exposure seem to reduce the beneficial properties of this compound. This effect, being slightly visible in Nrf2, is insufficient to mask RS-GRA action on the redox pathway. On the other hand, it may be enough to mask its action on the necroptosis pathway, which may explain why RS-GRA correlation with necroptosis has not been previously described in the literature.

### Strenghts and Limits

This work’s strengths are mainly related to the transcriptomic analysis methodology, which provides the investigation of genes involved in many different pathways. This approach allows for the simultaneous examination of multiple genes, permitting a more complete and exhaustive evaluation of intricate biological processes. Also, the rigorous filtering we applied to the data would be helpful to reduce false positives and would ease the identification of core processes related to the action of RS-GRA. The principal limitation of this study is that it has an exploratory purpose: while we were able to pinpoint pathways of interest, specifically aimed studies are needed to validate and better characterize our results. For instance, the dose effect could differ from an in vitro to an in vivo model. Indeed, although an in vitro model can simplify the evaluation of the compound on target cells, we were not able to assess the kinetics of the molecule, which could result in alterations of its concentration in the acting site (e.g., brain). Furthermore, an in vitro model does not account for potential alternative metabolic pathways to which the compound may be exposed. Finally, the precise molecular mechanism of action of RS-GRA has not been fully elucidated in this study.

## 5. Conclusions

From our results, we observed that RS-GRA presents a dual action: on the one hand it triggers the activation of the antioxidant response, heightening the expression of several detoxification and antioxidant enzymes; on the other hand, the compound seems to downregulate the necroptotic pathway, which, as discussed before, seems to be strictly associated with several neurodegenerative pathologies. As such, we can hypothesize that the compound may prove useful as potential adjuvant in pathological states where inflammation and cell death pathways are strongly activated, including neurodegenerative disorders. In this sense, it may be helpful not only to slow the progress of cognitive symptoms but also to aid in the rehabilitation of cognitive impairments. Moreover, based on the diverse effects observed at different dosages and time points, we can infer that the action of RS-GRA is influenced by both dosage and exposure duration, especially affecting the necroptosis pathway. Thus, the choice of dosage would be pivotal for the use of this substance as supplement. Acute supplementation with higher dosages would produce an acute effect at an earlier time point, with the risk of nullifying its action after prolonged exposure. A chronic supplementation would be manageable with lower dosages, which would avoid triggering the rapid increase observed at the higher concentrations. Overall, acute exposure would be selected in the 5–10 µM range, while chronic exposure could range between 1–5 µM.

It should be noted, though, that an effective application of our findings in a clinical setting would need confirmation from future in vivo studies, as the present investigation is unable to provide data on posology/kinetics of the compound in the organism. Furthermore, although no adverse effects are usually associated to RS-GRA in the literature, more specific investigations are needed to ensure the security of this compound. Future experiments using in vivo models are needed to better characterize this compound actions, the systemic distribution, and the overall kinetics within the organism.

## Figures and Tables

**Table 1:** Significantly perturbed pathways from SPIA.

Pathway.	pSize	NDE	pNDE	tA	pPERT	pG	pGFDR	pGFWER	Status
**KEGG**									
Necroptosis	111	3	2.07 × 10^−3^	−2.95	1.20 × 10^−2^	1.45 × 10^−4^	4.64 × 10^−3^	4.64 × 10^−3^	Inhibited
**PathBank**									
Ubiquitin–proteasome pathway	27	1	6.00 × 10^−2^	−5.65 × 10^−2^	1.58 × 10^−1^	3.54 × 10^−2^	3.54 × 10^−2^	3.54 × 10^−2^	Inhibited
**WikiPathways**									
Oxidative stress and redox pathway	65	2	9.68 × 10^−3^	3.17	1.60 × 10^−3^	9.28 × 10^−5^	1.11 × 10^−3^	1.11 × 10^−3^	Activated
Prostaglandin synthesis and regulation	11	2	2.77 × 10^−4^	−5.05 × 10^−1^	1.59 × 10^−1^	8.23 × 10^−4^	4.94 × 10^−3^	9.88 × 10^−3^	Inhibited
One-carbon metabolism and related pathways	32	1	7.07 × 10^−2^	7.92 × 10^−1^	7.20 × 10^−3^	2.80 × 10^−3^	1.12 × 10^−2^	3.36 × 10^−2^	Activated

In **Table 1**, we report the pathways significantly enriched and significantly perturbed according to our analysis. **pSize** = the number of genes in the pathway; **NDE** = number of differentially expressed genes in the pathway; **pNDE** = hypergeometric probability of observing Nde differentially expressed genes in the pathway by chance; **tA** = observed value of the perturbation score; **pPERT** = bootstrap probability associated to tA; **pGFDR** = adjusted pG using false discovery rate correction; **pGFWER** = adjusted pG using family-wise error rate (Bonferroni); **Status** = inhibition/activation according to the negative/positive sign of tA.

## Data Availability

The data supporting the findings of this study are openly available in the NCBI Sequence Read Archive at BioProject, accession number PRJNA1117421.

## Supplementary Materials

The following supporting information can be downloaded at: https://www.mdpi.com/article/10.3390/antiox13091111/s1, Figure S1: SPIA results for necroptosis and oxidative stress and redox pathway; Figure S2: Ripk1 WB data; Figure S3: Mlkl WB data; Figure S4: Ripk3 WB data; Figure S5: Nrf2 WB data; Figure S6: Concentration-related and time-related Sod1 changes; Figure S7: Sod1 concentrations across all conditions; Figure S8: Sod1 WB data.
