# Supplementary material for: Bioactivated Glucoraphanin Modulates Genes Involved in Necroptosis on Motor-Neuron-like Nsc-34: A Transcriptomic Study
Source: Antioxidants (Basel). 2024 Sep 14;13(9):1111. doi: 10.3390/antiox13091111 (PMC11428517; doi:10.3390/antiox13091111)
Supplement: Supplementary file 1 [file antioxidants-13-01111-s001.zip › antioxidants-3164562-supplementary.pdf]

Supplementary Data

Plot data from SPIA analysis on the enriched pathways

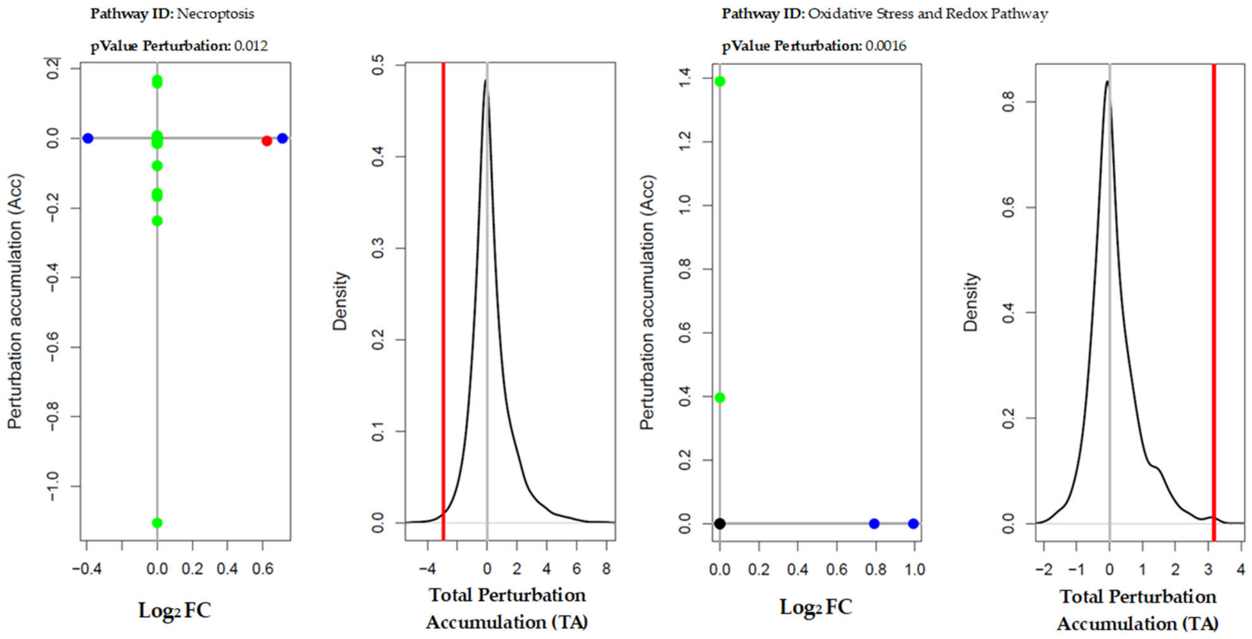

Supplementary Figure S1. The plots show the total perturbation detected on the two enriched pathways according to SPIA analysis. On the left-hand side we report the perturbation plot for Necroptosis. On the right-hand side we report the plot for oxidative Stress and Redox Pathway

Western Blot results for the investigated proteins

In the following section we report the Western blot gels for each investigated protein

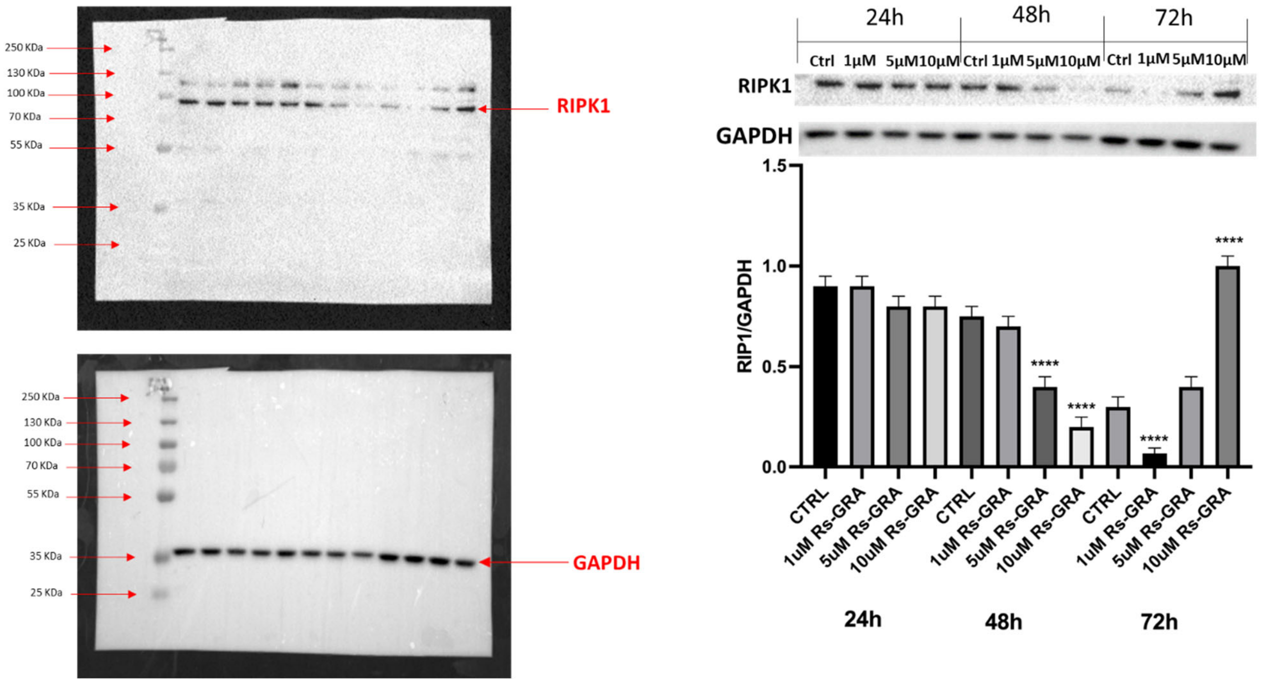

Supplementary Figure S2. Here, we report the results obtained from western blot analysis of Ripk1 expression evaluated on the cytosolic protein fraction. On the left-hand side of the figure, we report the original pictures of the membranes of Ripk1 and Gapdh. Gapdh was used as housekeeping protein to normalize the absorbance values of Ripk1 at the various tested conditions. For Ripk1 a significant decrease of protein expression compared to controls was highlighted at the later time-steps (48h at 5 µM and 10 µM dosages and 72h at 1 µM). Interestingly, we detected a drastic increase of this protein expression at 72h at 10 µM dosage. This result suggests the existence of a complex regulating mechanism that takes into consideration both concentration and exposure duration. The exposition to RS-GRA does indeed have down-regulatory effect on Ripk1, however, the prolonged exposition (more than 48h) may trigger a counterbalancing mechanic which in turn increase Ripk1 expression. This effect is visible especially on higher concentrations, while does not seem to be triggered if cells are exposed to low concentrations of RS-GRA (72h at 1 µM). a potential delayed effect of RS-GRA. Lower concentrations appear to have a negligible effect on this protein expression.

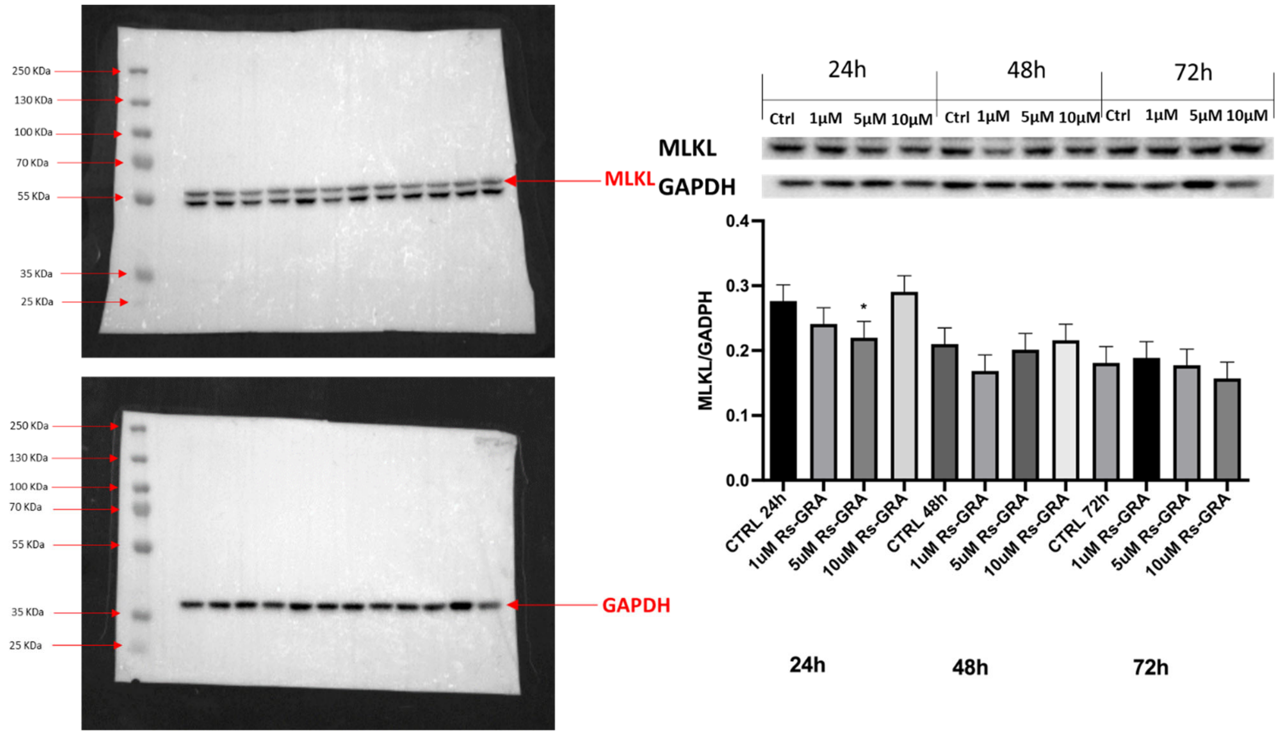

Supplementary Figure S3. Here, we report the results obtained from western blot analysis of Mlkl expression evaluated on the cytosolic protein fraction. On the left-hand side of the figure, we report the original pictures of the membranes of Mlkl and Gapdh. Gapdh was used as housekeeping protein to normalize the absorbance values of Mlkl at the various tested conditions. For Mlkl a significant decrease of protein expression compared to controls was highlighted only at the 5 µM at 24h. Overall, these results suggest that the action of RS-GRA at the transcriptomic level may be counterbalanced by other mechanisms (i.e. decreased degradation) which ultimately result in no significant perturbations of Mlkl levels. RS-GRA action may be mainly linked to other elements of the necroptosis pathway.

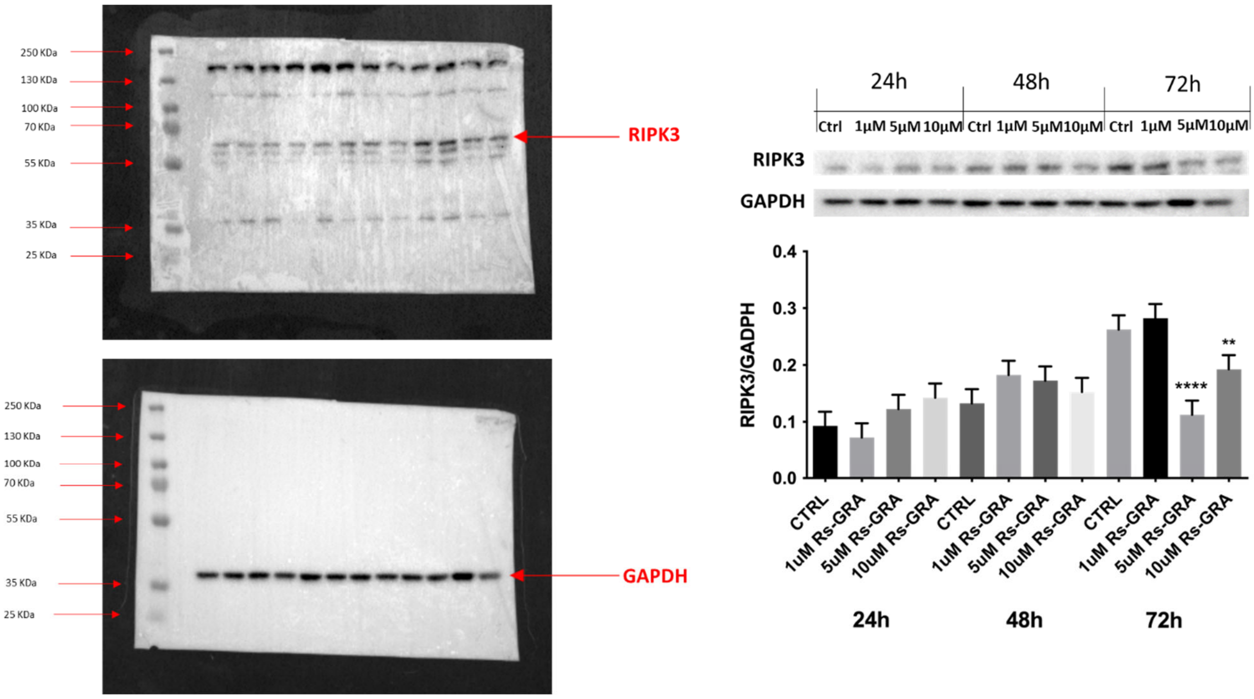

Supplementary Figure S4. Here, we report the results obtained from western blot analysis of Ripk3 expression evaluated on the cytosolic protein fraction. On the left-hand side of the figure, we report the original pictures of the membranes of Ripk3 and Gapdh. Gapdh was used as housekeeping protein to normalize the absorbance values of Ripk3 at the various tested conditions. For Ripk3 a significant decrease of protein expression compared to controls was highlighted at the last time-step (72h) at 5 µM and 10 µM dosages. This result suggests a potential delayed effect of RS-GRA. Lower concentrations appear to have a negligible effect on this protein expression.

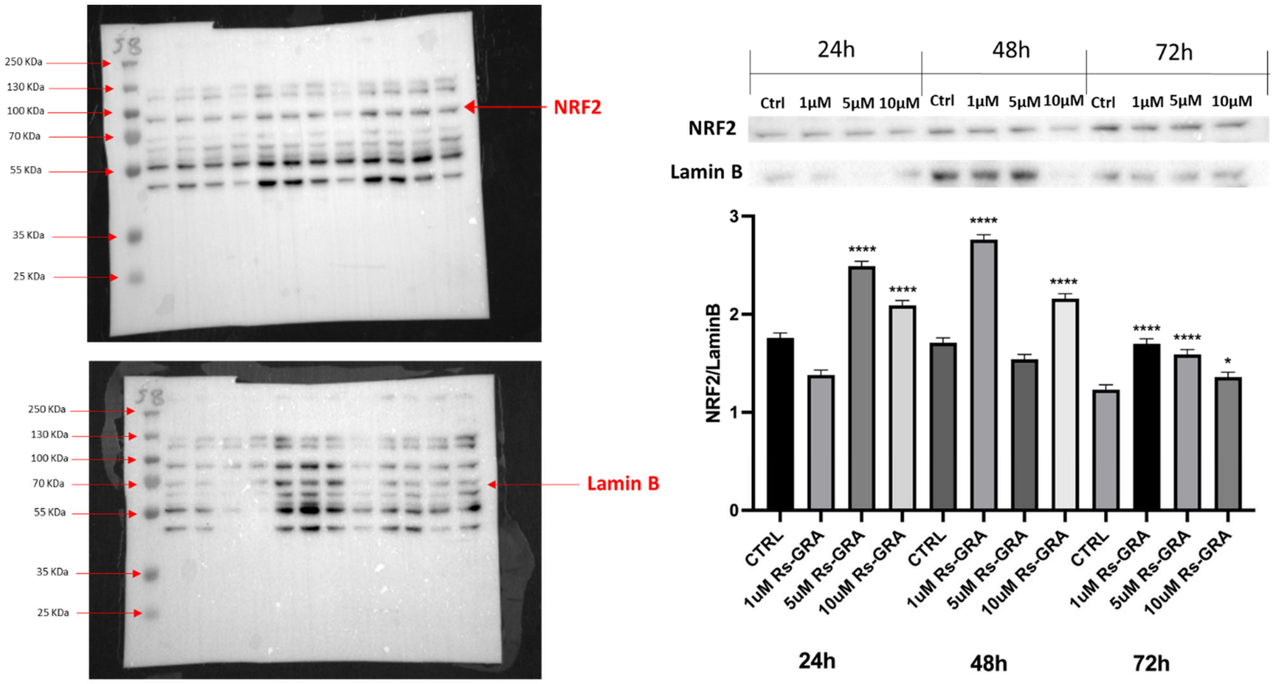

Supplementary Figure S5. Here, we report the results obtained from western blot analysis of Nrf2 expression evaluated on the nuclear protein fraction. On the left-hand side of the figure, we report the original pictures of the membranes of Nrf2 and Lamin B. Lamin B was used as housekeeping protein to normalize the absorbance values of Nrf2 at the various tested conditions. For Nrf2 a significant increase of protein expression compared to controls was observable at multiple time-steps and multiple dosages. Overall, these results suggest that the action of RS-GRA increases the nuclear concentration of Nrf2, a result in line to literature data and to the function of Nrf2. Indeed, this protein acts as a transcription factor that promotes the transcription anti-oxidant genes. To carry out its function, Nrf2 should be localized in the nucleus, for this reason we evaluated its expression in the nuclear protein fraction.

Sod1 WB data

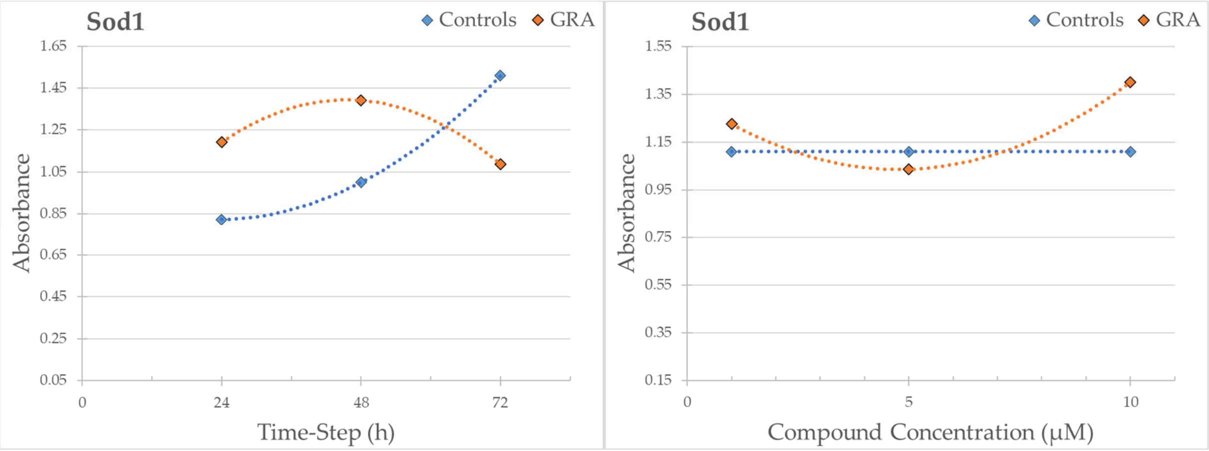

Supplementary Figure S6. On the left-hand side, we report the overall trend of proteins' concentrations at the three time-steps (24h, 48h, 72h). Each point corresponds to the mean protein expression (normalized by the housekeeping protein expression) of the three dosages for each time-step. On the right-hand side, we report the overall trend of proteins' concentrations at the three tested concentrations (1μM, 5μM and 10μM) irrespective of the time-steps. Each point corresponds to the mean protein expression (normalized by the housekeeping protein expression) of the three time-steps for each dosage. In orange the protein expression levels from treated cells are reported. In blue the non-treated controls.

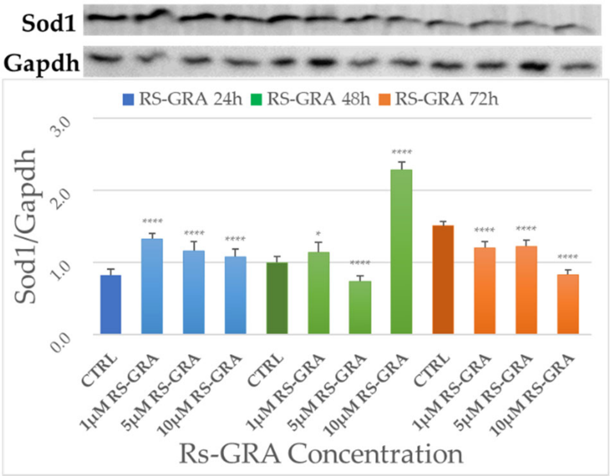

Supplementary Figure S7. Here, we report the concentration of proteins levels at all concentrations and all time-steps (bar plots) and the bands from WB membranes. Asterisks (*) indicate p-value: * $p < 0.05$; ** $p < 0.01$; *** $p < 0.001$; **** $p < 0.0001$, respectively.

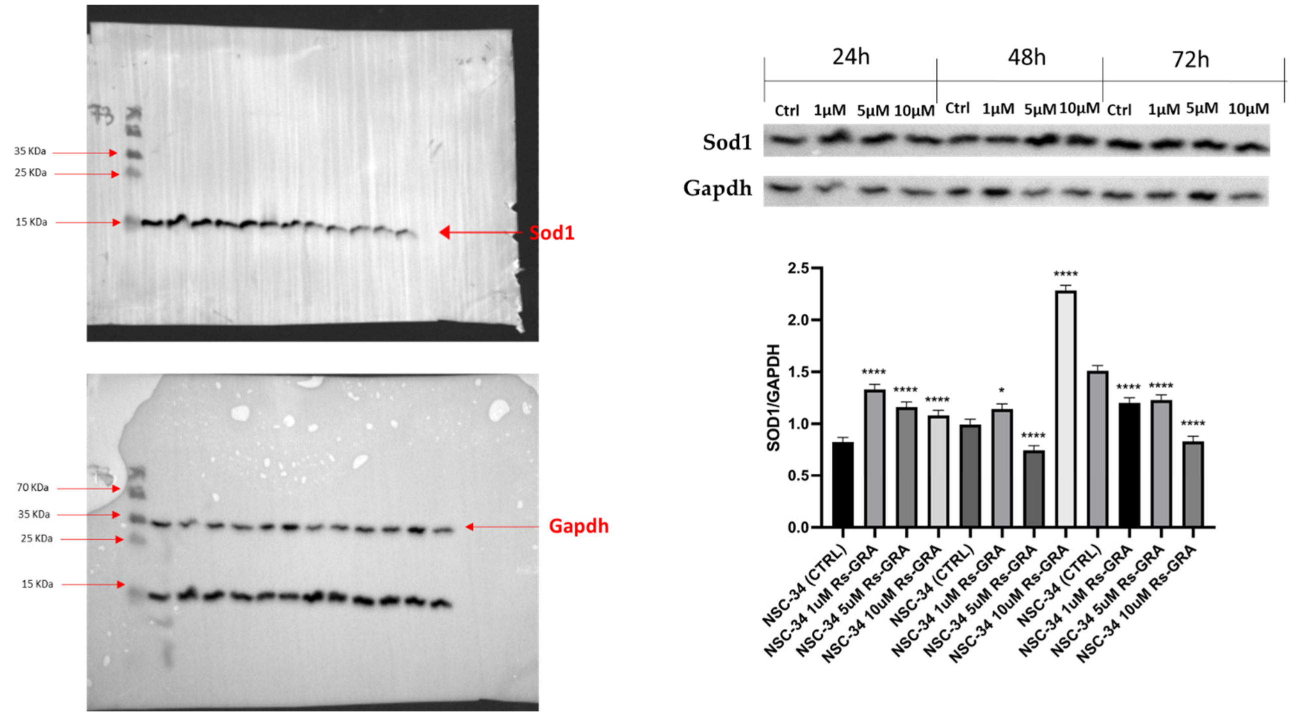

Supplementary Figure S8. Here, we report the results obtained from western blot analysis of Sod1 expression evaluated on the cytosolic protein fraction. On the left-hand side of the figure, we report the original pictures of the membranes of Sod1 and Gapdh. Gapdh was used as housekeeping protein to normalize the absorbance values of Sod1 at the various tested conditions. For Sod1 a significant decrease of protein expression compared to controls was highlighted at the last time-step (72h) at 10 μM dosages. This result suggests a potential delayed effect of RS-GRA. Lower concentrations appear to have a negligible effect on this protein expression.
